# In Vitro Activity of Essential Oils Distilled from Colombian Plants against *Candida* *auris* and Other *Candida* Species with Different Antifungal Susceptibility Profiles

**DOI:** 10.3390/molecules27206837

**Published:** 2022-10-12

**Authors:** Carolina Zapata-Zapata, Manuela Loaiza-Oliva, María C. Martínez-Pabón, Elena E. Stashenko, Ana C. Mesa-Arango

**Affiliations:** 1 Grupo de Investigación Dermatológica, Universidad de Antioquia, Medellín 050010, Colombia; 2 Grupo de Investigación en Patología Oral, Periodoncia y Cirugía Alveólo-Dentaria, Universidad de Antioquia, Medellín 050010, Colombia; 3 CROM-MASS-CENIVAM-Universidad Industrial de Santander, Bucaramanga 68002, Colombia

**Keywords:** *Candida* *auris*, *Candida* spp., *Lippia* spp., antifungal agents, essential oils, MIC, cytotoxicity, time–kill assays

## Abstract

Multi-drug resistant species such as *Candida auris* are a global health threat. This scenario has highlighted the need to search for antifungal alternatives. Essential oils (EOs), or some of their major compounds, could be a source of new antifungal molecules. The aim of this study was to evaluate the in vitro activity of EOs and some terpenes against *C. auris* and other *Candida* spp. The eleven EOs evaluated were obtained by hydro-distillation from different Colombian plants and the terpenes were purchased. EO chemical compositions were obtained by gas chromatography/mass spectrometry (GC/MS). Antifungal activity was evaluated following the CLSI standard M27, 4th Edition. Cytotoxicity was tested on the HaCaT cell line and fungal growth kinetics were tested by time–kill assays. *Candida* spp. showed different susceptibility to antifungals and the activity of EOs and terpenes was strain-dependent. The *Lippia origanoides* (thymol *+ p*-cymene) chemotype EO, thymol, carvacrol, and limonene were the most active, mainly against drug-resistant strains. The most active EOs and terpenes were also slightly cytotoxic on the HaCaT cells. The findings of this study suggest that some EOs and commercial terpenes can be a source for the development of new anti-*Candida* products and aid the identification of new antifungal targets or action mechanisms.

## 1. Introduction

In recent decades, there has been a notorious increase in infections caused by naturally resistant *Candida* spp. or by strains that have developed resistant phenotypes during treatment [1]. *Candida albicans*, *C. tropicalis*, *C. parapsilosis*, and *C. glabrata* are still the most frequent species causing infections. However, multi-resistant emerging *Candida* spp., such as *C. auris*, have been increasingly reported. *C. auris* is associated with high morbidity and mortality rates, almost exclusively in hospital settings [2]. The management of these infections is challenging due to the resistance of *C. auris* to several antifungals [3,4,5]. Additionally, these yeasts have the ability to form biofilms on medical devices and biomaterials, such as catheters and heart valves [1]. *C. auris* can also persist in hospital environments causing outbreaks, mainly in intensive care units (ICU) [2]. The selection of resistant isolates of different *Candida* spp. and the emergence of pan-resistant or multi-resistant species threaten the future management of fungal infections due to the scant antifungal options for clinical use [5,6]. Moreover, most antifungal drugs have a narrow spectrum and cause considerable side-effects [7]. This scenario has increased the amount of interest in exploring new molecules targeting different cellular components.

Natural sources, such as plant extracts and essential oils (EOs), may be effective alternatives in the search for new antifungal agents [8,9]. EOs are volatile secondary metabolites distilled from aromatic plants and are molecules of different chemical natures, mostly terpenoids and phenylpropanoids [10]. The antifungal properties of different EOs, as well as their compounds, have been previously demonstrated [11].

Colombia is among the top ten most biodiverse countries in the world and ranks second in plant diversity. In 2020 there were 30,014 recorded plant species, of which 6499 were endemic [12]. The enormous biodiversity of Colombia becomes an invaluable source of natural bioactive compounds [13], within which it may be possible to find some active compounds against clinically relevant fungi, including multi-drug or pan-drug resistant species. *Lippia origanoides* and *L. alba* (both from the Verbenaceae family), growing in various countries of South America, including Colombia [12,13], are some of the most studied neo-tropical plants due to the different biological activities that their EOs have displayed, including antifungal properties [14]. 

Two chemotypes of *L. alba* growing in Colombia have been identified: the (citral + caryophyllene oxide) chemotype and the (carvone + limonene) chemotype, named by their major EO compounds [15,16]. The anti-inflammatory and antimicrobial activities of these EOs, some of their enriched fractions, and commercial terpenes have previously been studied [15,17].

This study focuses on investigating the in vitro effects of EOs distilled from Colombian plants of the Verbenaceae family, and some of their major compounds, against clinical isolates of *C. auris* and other *Candida* spp. with different antifungal susceptibility to the most common antifungal drugs. The chemical composition, cytotoxicity, and fungal growth kinetics of the most active EOs and commercial terpenes were evaluated.

## 2. Results

### 2.1. Essential Oil Composition

Eleven EOs distilled from different *L. origanoides* chemotypes (*L. alba*, *Varronia curassavica*, *Piper marginatum*, *Ageratina* cf. *popayanensis*, and *Pogostemon cablin*) were chemically characterized by GC/MS. Information regarding the plants from which the most active EOs were obtained, plant chemotypes, and EO chemical compositions are shown in Table 1.

*Candida* spp. strains and clinical isolates displayed different antifungal susceptibility profiles. The minimal inhibitory concentration (MIC) values for amphotericin B (AMB), fluconazole (FLC), itraconazole (ITC), and caspofungin (CSF) are presented in Table 2. The azole and AMB resistance of *C. tropicalis* ATCC 200956 was confirmed, as well as the resistance to CSF and FLC of *C. glabrata* LMDM 34 and *C. parapsilosis* Synlab 406, respectively. The susceptibility of *C. auris* was strain-dependent. High MIC values to FLC and AMB were obtained. According to the Centers of Disease Control and Prevention (CDC) breakpoints [18], *C. auris* Ca 41, *C. auris* Ca 45, and *C. auris* Ca 46 were considered AMB-resistant (MIC range 1–2 µg/mL) and *C. auris* Ca 17 FLC-resistant (MIC = 32 µg/mL).

### 2.2. Antifungal Activity of Essential Oils and Terpenes

**Figure 1:**
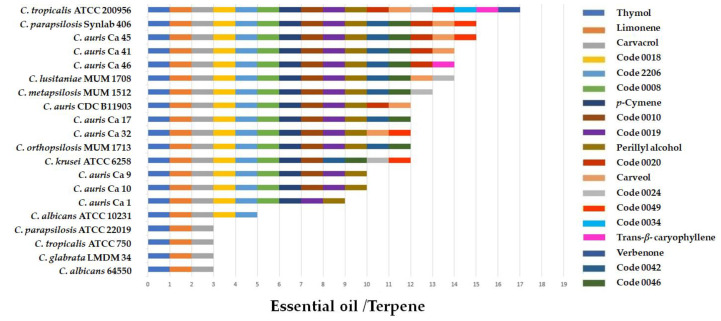
Results of screening the in vitro activity of EOs and some commercial terpenes against different *Candida* species. The presence of the bar indicates antifungal activity at 256 µg/mL.

The results of the screening of the eleven EOs and the eight commercial terpenes at 256 µg/mL against the twenty *Candida* spp. strains are shown in Figure 1. Antifungal activity was strain-dependent. Limonene, thymol, and carvacrol were active against all tested *Candida* spp. Perillyl alcohol and *p*-cymene were active against 90% and 100% of *C. auris*, respectively, while verbenone, carveol, and trans-β-caryophyllene were active against only some of the clinical isolates of *C. auris*. The EOs of the *L. origanoides* (thymol + *p*-cymene) chemotype (Code 0018) and of the *L. origanoides* (carvacrol + thymol) chemotype (Code 2206) were active against 80% of the tested yeasts. The EOs of the *L. origanoides* carvacrol chemotype (Code 0008) and *L. origanoides* thymol (Codes 0019 and 0010) chemotypes inhibited 75% and 70% of the yeasts evaluated, respectively. The EOs distilled from *Lippia micromera* (Code 0020), *P. marginatum* (Code 0024), *A*. cf. *popayanensis* (Code 0034), *Verronia curassavica* (Code 0042), the *L. alba* citral chemotype (Code 0046), and *P. cablin* (Code 0049) were less active (Figure 1). 

For the most active EOs and commercial terpenes, minimal inhibitory concentrations (MICs) were determined. Important results were observed with the resistant strains *C. tropicalis* ATCC 200956 and *C. parapsilosis* Synlab 406. MIC values for non-*C. auris* species are shown in Table 3.

We separately analyzed results obtained with the emergent yeast *C. auris.* MIC values for EOs, and some commercial terpenes, are shown Table 4. As for the other species, the antifungal activity of EOs and some commercial terpenes was strain-dependent. The best activity was observed with limonene (MIC range 16–64 µg/mL).

### 2.3. Cytotoxic Activity 

The cytotoxicity of the EOs and commercial terpenes that showed the highest antifungal activity was evaluated by MTT assay on the immortalized human keratinocytes cell line (HaCaT). The 50% cytotoxic concentrations (CC_50_) and the selectivity index (SI) values are shown in Table 5. The less cytotoxic EOs corresponded to the *L. origanoides* thymol chemotype (Code 0010), the *L. origanoides* (carvacrol + thymol) chemotype (Code 2206), the *L. origanoides* (carvacrol + *p*-cymene) chemotype (Code 0008), and the *L. origanoides* (thymol + *p*-cymene) chemotype (Code 0018). CC_50_ values were 903.6, 788.0, 877.9, and 665.9 µg/mL, respectively. The SI values were strain-dependent, and the highest SI values were observed with resistant strains or strains that had high MICs towards antifungals but were sensitive to EOs or commercial terpenes.

### 2.4. Time–Kill Assays

**Figure 2:**
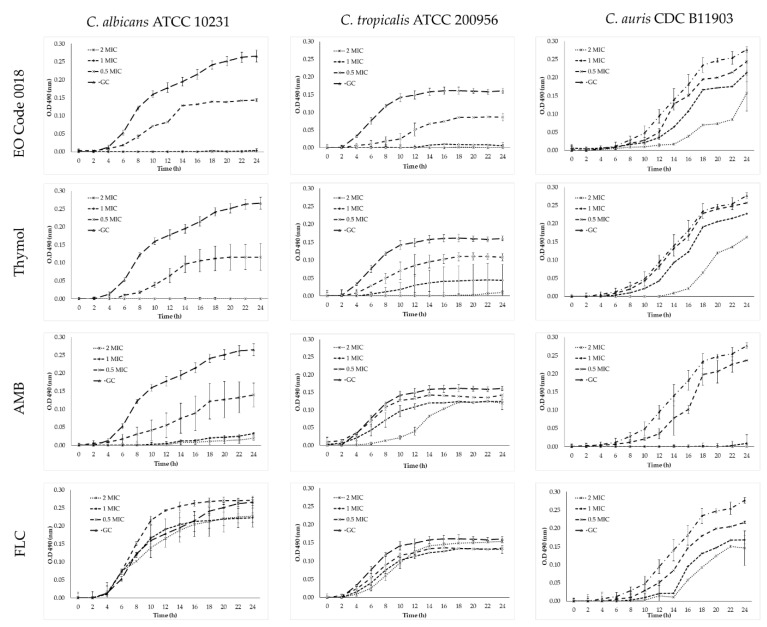
Time–kill curve plots for *C*. *albicans* ATCC 10231, *C. tropicalis* ATCC 200956, and *C. auris* CDC B11903 in the presence of the *L. origanoides* (thymol + *p*-cymene) chemotype (Code 0018) EO, thymol, AMB, and FLC.

Plots of the activity of the *L. origanoides* (thymol + *p*-cymene) chemotype (Code 0018) EO, thymol, and the antifungals AMB and FLC against *C. albicans* ATCC 10231, *C. tropicalis* ATCC 200956, and *C. auris* CDC B11903 are shown in Figure 2.

Both the *L. origanoides* (thymol + *p*-cymene) chemotype (Code 0018) EO and the commercial terpene thymol showed fungicidal effects at 1X and 2X MIC against *C. albicans* ATCC 10231 and *C. tropicalis* ATCC 200956. On the other hand, *C. auris* CDC B11903 growth was not affected, and an extended lag-phase was observed at 2X MIC. Additionally, the expected fungistatic and fungicidal activities of FLC and AMB, respectively, were demonstrated.

## 3. Discussion

Fungal infections are increasing at an alarming rate in parallel with the occurrence of infections caused by antifungal-resistant strains. The morbidity and mortality of these infections have led researchers to seek options for the development of new, less-toxic antifungal agents with new targets or mechanisms of action [7,19]. 

EOs have been considered as promising agents for their antimicrobial activity [10]. In recent decades, several studies have demonstrated the antifungal activity of EOs, and some of their compounds, against fungi of clinical and agricultural importance (*Candida* spp. and filamentous fungi) [9,20,21,22,23,24,25,26,27,28]. These antifungal activities have been attributed to either the synergy between the multiple compounds that constitute EOs, or the major components of EOs [10,29].

This study showed that the EOs distilled from the *L. origanoides* thymol (Codes 0010 and 0019) chemotype and from *L*. *origanoides (*thymol + *p*-cymene) chemotype (Code 0018) were the most active against different *Candida* spp. (Table 3 and Table 4). The EO activity can be attributed to the major compounds (thymol, carvacrol or *p*-cymene). 

Currently, there are no reference protocols for evaluating the in vitro antifungal activity of natural compounds. Therefore, there is a great variability in the scientific literature about the techniques used and concentrations tested [9,22,25,30,31]. The evaluation of anti-*Candida* activity for the EOs and commercial terpenes in this study was performed with the standard CLSI M27 technique, which was designed for the evaluation of antifungals for clinical use, with some adjustments [32]. 

To define the antifungal activity of the different samples, we categorized the activity according to Holetz et al. [33] as follows: MIC values of ≤100 μg/mL were classified as having good activity, values of >100 and ≤500 μg/mL were classified as moderate, and values of >500 μg/mL were classified as weak activity. In agreement with these criteria, it was possible to identify good activity for some EOs and commercial terpenes; the highest activity was observed for the monoterpene limonene (MIC range 16–64 μg/mL) (Table 3 and Table 4). 

We separately analyzed results obtained with the emergent yeast *C. auris.* MIC values for EOs, and some commercial terpenes, are shown in Table 4. As for the other species, the antifungal activity of EOs and some commercial terpenes was strain-dependent. The best activity was observed with limonene (MIC range 16–64 µg/mL).

We found that the strains resistant to the main antifungal agents in clinical use (*C. tropicalis* ATCC 200956, *C. parapsilosis* Synlab 406, and *C. auris*) were the most susceptible to the studied EOs and commercial terpenes (Table 3 and Table 4). These findings suggest that these compounds have different targets and/or mechanisms of action to those described for conventional antifungals in clinical use [34]. 

In addition, considering that the cross-resistant *C. tropicalis* ATCC 200956 strain harbors a deletion of 132 bp in the *ERG11* gene, a mutation in the *ERG3* gene, and the lack of ergosterol in the membrane [35], it is possible that the activity of the EOs and terpenes was not related to the main azoles or AMB targets. However, the fact that few EOs and terpenes showed activity against *C. glabrata* LMDM 34 (echinocandin-resistant strain harbouring a substitution at the Fks2p subunit of the *β*-D-1,3-glucan synthase catalytic complex, the target of these lipopeptides) [36,37] suggests that the aforementioned compounds may act on fungal cell wall synthesis or its structure. This assumption can be supported by the results obtained by Brennan et al. [38]. They demonstrated that limonene inducted the expression of *Saccharomyces cerevisiae* genes linked to the organization and biogenesis of the cell wall. Additionally, there were no changes in the compounds and characteristics of the lipid membrane (fluidity, fatty acids, ergosterol, and saturated or unsaturated fatty biosynthesis pathways had not changed). Further, bearing in mind that *FKS2* expression is dependent upon the calcium/calcineurin/Hsp90 signaling pathway [37,39], the activity of the EOs and commercial terpenes studied could be associated with the same pathway as well [40]. Further work is needed to elucidate the targets or mechanisms of action of these EOs and terpenes. 

Concerning *C. auris*, few studies have examined the in vitro activity of the EOs and terpenes studied here against this species. Recently, Baldim et al. [41] reported the anti-*C. auris* activity of *L. sidoides* EO (MIC range 140–563 μg/mL). Shaban et al. [4] reported a moderate activity of carvacrol (MIC range 63–250 μg/mL) and thymol (MIC range: 156–625 μg/mL). These results were similar to those obtained in this study. It is worth mentioning that this is the first study which describes the anti-*C. auris* activity of limonene, *p*-cymene, and of the *L. origanoides* EOs. *C. auris* can produce biofilms on hospital surfaces and medical devices or colonize healthcare personnel [2,6]; therefore, the EOs and commercial terpenes studied could be promising options for disinfection and/or decontamination of hospital surfaces and environments [29].

Other studies have also evaluated the cytotoxicity of the EOs distilled from plants not belonging to the genus *Lippia* and from the terpenes studied here on the HaCaT cell line, with CC_50_ values ranging between 33.93 and 1701.97 μg/mL [42,43,44]. The CC_50_ values obtained in our study with the *Lippia* spp. EOs and commercial terpenes ranged between 354.7 and 903.6 μg/mL (Table 5). In general, the EOs were less cytotoxic than the commercial terpenes, presumably due to the interactions among the EO compounds, which can decrease their cytotoxicity. The CC_50_ values obtained in this study for the EOs of *L. origanoides*, limonene, carvacrol, and thymol were higher compared to those published by other authors [45,46,47], indicating that the EOs studied were less cytotoxic. 

The low toxicity and preference of the EOs for fungal cells are ideal characteristics for the development of new antifungals. Interestingly, both the *L. origanoides* (thymol + *p*-cymene) chemotype (Code 0018) EO and thymol could be adjusted to those characteristics. These samples were selected to perform time–kill assays to define whether the effect was fungicidal or fungistatic. Figure 2 shows that the *L. origanoides* (thymol + *p*-cymene) chemotype (Code 0018) EO and thymol were fungicidal against *C. albicans* ATCC 10231 and *C. tropicalis* ATCC 200956 at 1X and 2X MIC. Oppositely, these compounds did not show fungicidal activity at any concentration against *C. auris* CDC B11903, even when MIC values were low. This *C. auris* behavior could possibly be explained by a quorum sensing effect promoted by the higher inoculum size used in the time–kill assays (5 × 10^5^ CFU/mL) compared to that used for the MICs (0.5–5 × 10^3^ CFU/mL). 

The data obtained in this study could be the starting point for further research aimed at the development of topical or antiseptic products against resistant *Candida* spp. based on aromatic and medicinal Colombian plants. In the future, models such as the one proposed by Rayan et al. and Masalha et al. [48,49] could be applied to confirm whether the results obtained in this in vitro study could be confirmed with this predictive model. It would also be of interest to carry out an analysis via molecular docking in order to obtain information on the bioactivity mechanism of the most active terpenes or of those components of the active EOs identified in this study.

## 4. Materials and Methods

### 4.1. Plant Materials and Essential Oil Distillation

All plants used in this work were cultivated under controlled agricultural conditions in the experimental plots of the garden of the National Center for Agroindustrialisation of Aromatic and Medicinal Tropical Plant Species (CENIVAM) at the Industrial University of Santander (UIS, Bucaramanga, Colombia). The taxonomic identification was performed at the Colombian National Herbarium (National University of Colombia, UN, Bogotá, Colombia) and at the UIS Herbarium. The exsiccatae and vouchers were placed at the UIS Herbarium. The EOs were distilled from different chemotypes of L. origanoides (Codes 2206, 0008, 0010, 0018, and 0019). The voucher numbers of these plants are shown in Table 1. EOs were also distilled from the following plants: the *L. alba* citral chemotype (Code 0046; 22002 UIS Herbarium), *L. micromera* (Code 0020, sample in Herbarium), *V. curassavica* (Code 0042; 20892 UIS Herbarium), *P. marginatum* Jacq (Code 0024; 21966 UIS Herbarium), *A*. cf. *popayanensis* (Hieron) R. King & H. Rob (Code 0034; 22040 UIS Herbarium), and *P. cablin* (Code 0049; 20890 UIS Herbarium). Plants were initially collected in the countryside in Barbosa, Betulia or in San Vicente de Chucurí (Santander, Colombia), propagated, and grown in the CENIVAM experimental plots. The mean environment temperature varied between 26 and 29 °C, with relative humidity of 75–80%. The plants were gathered in their flowering stages and only undamaged aerial plant parts were used for EO extraction by hydro-distillation. The hydro-distillation was carried out immediately after the vegetable material was harvested, without its previous drying or weathering. EOs were distilled (2 h) from fresh plant material (500 g) on a Clevenger apparatus. The EOs were dried using anhydrous sodium sulphate and kept under nitrogen atmosphere at 4 °C in darkness.

### 4.2. Sample Preparation 

Each EO was weighed (50 mg) and dissolved in 1 mL of CH_2_Cl_2_; an aliquot of this dilution (2 µL) was injected into a gas chromatograph (GC) coupled to a mass selective (MS) or flame ionization (FID) detection system.

### 4.3. Chromatographic Analysis

Analysis was performed on a GC 6890 Plus gas chromatograph (Agilent Technologies, AT, Palo Alto, CA, USA) equipped with a mass selective detector MS 5973 Network (AT, Palo Alto, CA, USA) using electron ionization (EI, 70 eV). Helium (99.995%, AP gas, Messer, Bogotá, Colombia) was used as a carrier gas, with initial inlet pressure at the head of the column of 113.5 kPa; the volumetric flow rate of the carrier gas during the chromatographic run was kept constant (1 mL/min). The injection mode was split (30:1) and the injector temperature was kept at 250 °C.

Compound separation was carried out on two capillary columns, one with the polar stationary phase of poly(ethylene glycol) (PEG) (DB-WAX, J & W Scientific, Folsom, CA, USA) of 60 m (L) × 0.25 mm (I.D.) × 0.25 μm (d_f_), and another with a non-polar stationary phase of 5%-phenyl-poly(methyl siloxane) (5%-Ph-PDMS) (DB-5MS, J & W Scientific, Folsom, CA, USA) of the same dimensions. In the polar column (DB-WAX), the oven temperature was programmed from 50 °C (5 min) to 150 °C (7 min), at 4 °C/min, and then up to 230 °C (50 min) at 4 °C/min. For the non-polar column (DB-5MS), the temperature of the chromatographic oven was programmed from 45 °C (5 min) to 150 °C (2 min), at 4 °C/min, then up to 300 °C (10 min) at 5 °C/min. The temperature of the GC-MS transfer line was set at 230 °C when the polar column was used and at 300 °C for the non-polar column. The temperatures of the ionization chamber and the quadrupole were 250 °C and 150 °C, respectively. The mass range for the acquisition of ion currents was m/z 45–450 u, with an acquisition speed of 3.58 scan/s. Data were processed with MSD ChemStation G1701DA software (AT, Palo Alto, CA, USA). The identification of compounds was carried out based on their linear retention indices (LRI), calculated from the retention times of the compound of interest and the C_6_–C_25_ and C_8_–C_40_ n-alkanes (Sigma-Aldrich, St. Louis, MO, USA) on both polar and non-polar capillary columns.

For EO component identification, the experimental mass spectrum of each compound was compared to that from QUADLIB-2007, NIST (2017), and Wiley (2008) spectral databases. Confirmatory identification of some compounds was made by comparison of their LRIs and mass spectra with those of available standard substances. For the quantitative analyses (relative amounts, %), the EO samples, prepared as described above, were injected into the GC 6890 Plus gas chromatograph (AT, Palo Alto, CA, USA) coupled to a flame ionization detection system (GC/FID) and a non-polar 5%-Ph-PDMS capillary column (DB-5MS, J & W Scientific, Folsom, CA, USA) of the same dimensions (L, I.D., d_f_) as that used for the GC/MS analysis. The GC-FID oven temperature was programmed in a similar manner as for the GC-MS equipment described previously; the EO samples were injected in split mode (30:1) and the temperatures of the injection port and the FID were maintained at 250 °C. 

### 4.4. Antifungals 

For antifungal profile identification of *Candida* species, amphotericin B (AMB), fluconazole (FLC), itraconzole (ITC), and caspofungine (CSF) (Sigma-Aldrich, St. Louis, MO, USA) were tested.

### 4.5. Essential Oils and Terpenes

The antifungal activity of eleven EOs and eight commercial terpenes was evaluated in vitro. EOs were distilled from five plants of different *L. origanoides* chemotypes (Codes 2206, 0008, 0010, 0018, and 0019), the *L. alba* citral chemotype (Code 0046), *L. micromera* (Code 0020), *V. curassavica* (Code 0042), *P. marginatum* (Code 0024), *A*. cf. *popayanensis* (Code 0034), and *P. cablin* (Code 0049). The terpenes tested were limonene (97%), carvacrol (98%), thymol (98.5%), *p*-cymene (99%), perillyl alcohol (96%), carveol, mixture *cis* and *trans* (≥95%), verbenone (≥99%), and trans-*β*-caryophyllene (98.5%) (Sigma-Aldrich, St. Louis, MO, USA). A stock solution of each sample was prepared in dimethyl sulfoxide (DMSO; Sigma-Aldrich, St. Louis, MO, USA). 

### 4.6. Fungi

The fungal strains used in this study were *C. albicans* ATCC 10231, *C. albicans* ATCC 64550, *C. parapsilosis* ATCC 22019, *C. tropicalis* ATCC 750, *C. tropicalis* ATCC 200956 (resistant to azoles and AMB) [35], *C. glabrata* LMDM 34 (resistant to echinocandins) [36], *C. metapsilosis* MUM 15.12, *C. orthopsilosis* MUM 17.13, *C. lusitaniae* MUM 17.08, *C. krusei* ATCC 6258 (*Issatchenkia orientalis* ATCC 6258), and *C. auris* CDC B11903. Additionally, nine clinical isolates were included: *C. parapsilosis* Synlab 406 (FLC-resistant) and eight *C. auris* isolates identified by MALDI-TOF MS (Bruker Daltonics, Bremen, Germany) according to Zhao et al. [50]. These yeasts were cultured on Sabouraud Dextrose Agar (SDA; Sigma-Aldrich, St. Louis, MO, USA) for 24 h at 35 °C. 

### 4.7. Antifungal Susceptibility Testing (Antifungals, EOs, and Terpenes)

Antifungal susceptibility testing was performed according to Clinical and Laboratory Standards Institute M27, 4th Edition (CLSI standard M27, 4th Edition) [32]. Some modifications were applied for evaluation of the antifungal activity of EOs and terpenes. Initially, a screening was carried out to determine the antifungal activity of the EOs and of commercial terpenes. Stock concentrations of EOs and terpenes were prepared at 512 µg/mL and an inoculum of 2.5 × 10^3^ CFU/mL of each yeast was prepared in RPMI 1640-MOPS (Sigma- Aldrich St. Louis, MO, USA). Thereafter, 100 µL of each stock solution of EOs or terpenes was dispensed in 96-well microdilution plates (Corning^®^, Costar^®^, NY, USA) and 100 µL of each yeast inoculum was added. Hence, the tested concentration of the EOs and terpenes was 256 µg/mL, and the concentration of the inocula was 1.25 × 10^3^ CFU/mL. Microdilution plates were incubated at 35 °C and, after 24 h, the presence or absence of growth was visually checked using a manual mirror viewer and then compared with the amount of growth in the control (no EO or terpene). Compounds able to inhibit yeast growth were tested to determine the minimal inhibitory concentration (MIC) as follows: 100 µL of 10 two-fold dilutions of the EOs or commercial terpenes was dispensed in 96-well microplates and the yeast inoculum was added. Final concentrations of the EOs or terpenes ranged from 0.5 µg/mL to 256 µg/mL. Microdilution plates were incubated at 35 °C for 24 h. MICs were visually determined at the lowest concentration that produced visual inhibition compared to the growth control. The assays were performed at least three times in duplicate on different days. The results were expressed as geometric means (GM) and ranges. As an antifungal susceptibility testing control, the activity of ITC and AMB against the reference strains *C. krusei* ATCC 6258 and *C. parapsilosis* ATCC 22019 was evaluated in all experiments following the CLSI standard M27, 4th Edition [32]. It was mandatory that the MIC values remained in the accepted range. 

### 4.8. Cytotoxicity of the Essential Oils and Terpenes

The cytotoxicity of the most active EOs and commercial terpenes studied was tested on a non-tumor keratinocytes HaCaT cell line. The non-tumor keratinocytes HaCaT cell line was derived from primary epidermal keratinocytes from normal human adult (HEKa) PCS-200-011TM and was obtained from Dr. Juan Carlos Gallego-Gómez (Molecular and Translational Medicine Group, Universidad de Antioquia). Cells were cultured in Dulbecco’s Modified Eagle Medium (DMEM; Sigma-Aldrich, St. Louis, MO, USA) supplemented with 10% fetal bovine serum (Invitrogen, Carlsbad, CA, USA), 1% penicillin, streptomycin, and neomycin (Invitrogen, Carlsbad, CA, USA), and 1% L-glutamine (Invitrogen, Carlsbad, CA, USA) at 37 °C in a humidified atmosphere of 5% CO_2_. Once the confluence of the cells reached 80%, the cells were dissociated using trypsin (Sigma-Aldrich, St. Louis, MO, USA) and subcultured in 96-well microplates at a density of 1.6 × 10^4^ cells per well at 37 °C for 24 h with 5% CO_2_. Subsequently, the cells were treated with concentrations of selected compounds in a range between 0.25 and 2000 μg/mL at 37 °C for 24 h with 5% CO_2_. Afterwards, the culture medium was removed and 3-(4.5-dimethylthiazol-2-yl)-2.5-diphenyltetrazolium bromide (MTT) (Sigma-Aldrich, St. Louis, MO, USA) at 5 mg/mL was added at 37 °C for 2 h. Subsequently, DMSO was added to dissolve the formazan crystals. Finally, the absorbances were measured with a Multiskan SkyHigh Microplate Spectrophotometer (Thermo Fisher Scientific, Waltham, MA, USA) at λ = 570 nm. The CC_50_ values were obtained by linear regression analysis with concentration–response curves, which were performed with absorbance data using GraphPad Prism software (Prism 9.3.0, San Diego, CA, USA). The assays were conducted at two separate times in triplicate. The data were expressed as means. Additionally, the SI values were calculated by dividing CC_50_ values by the MIC values. 

### 4.9. Time–Kill Assays

Time–kill assays with the *L. origanoides* (thymol + *p*-cymene) chemotype (Code 0018) EO, thymol, FLC, and AMB against *C. albicans* ATCC 10231, *C. tropicalis* ATCC 200956, and *C. auris* CDC B11903 were performed according to the protocol published by Klebser et al. [51] with some modifications. Strains were subcultured at 35 °C for 24 h on SDA; therefore, 100 µL of different compound concentrations (0.5, 1, and 2X MIC) and 100 µL of a suspension of 5 × 10^5^ CFU/mL were added in 96-well microplates and were incubated at 35 °C for 24 h. Absorbance at λ = 490 nm was measured every 2 h with a Multiskan SkyHigh Microplate Spectrophotometer. AMB and FLC were included as fungicidal and fungistatic control drugs, respectively.

## Figures and Tables

**Table 1:** Plant information, chemotypes, and chemical compositions of the most active EOs.

Code	Plant Species and Chemotype	Collection Site	Voucher Number	Principal Compounds
2206	*L. origanoides* (Carvacrol + thymol) chemotype	Barbosa—Santander, Colombia	COL 587104	**Carvacrol (34.9%), thymol (23.3%)**, *γ*-terpinene (11.1%), *p*-cymene (9.0%), *trans*-*β*-caryophyllene (5.0%), *α*-humulene (2.5%), *α*-terpinene (1.8%), *β*-myrcene (1.7%), thymyl methyl ether (1.6%), and carvacryl acetate (0.8%).
0008	*L. origanoides* (Carvacrol + *p*-cymene) chemotype	Bucaramanga—Santander, Colombia	22034 UIS Herbarium	**Carvacrol (35.0%), *p*-cymene (14.4%)**, thymol (8.0%), *γ*-terpinene (5.3%), *trans*-*β*-caryophyllene (4.4%), *β*-myrcene (2.4%), carvacryl acetate (2.0%), thymyl methyl ether (1.9%), *α*-terpinene (1.7%), and *α*-thujene (1.6%).
0010	*L. origanoides* Thymol chemotype	Bucaramanga—Santander, Colombia	22035 UIS Herbarium	**Thymol (75.3%)**, *trans*-*β*-caryophyllene (5.4%), carvacrol (4.9%), *α*-humulene (3.2%), *p*-cymene (2.3%), thymyl acetate (1.6%), thymyl methyl ether (1.3%), caryophyllene oxide (1.3%), and *trans*-*β*-bergamotene (1.0%).
0018	*L. origanoides* (Thymol + *p*-cymene) chemotype	Bucaramanga—Santander, Colombia	22039 UIS Herbarium	**Thymol (49.4%), *p*-cymene (19.1%)**, *γ*-terpinene (9.2%), *β*-myrcene (5.2%), *α*-terpinene (2.9%), carvacrol (2.7%), thymyl methyl ether (1.8%), *trans*-*β*-caryophyllene (1.6%), *cis*-*β*-ocimene (1.2%), and limonene (0.9%).
0019	*L. origanoides* Thymol chemotype	Bucaramanga—Santander, Colombia	22036 UIS Herbarium	**Thymol (71.7%)**, *p*-cymene (10.5%), carvacrol (4.4%), *β*-myrcene (2.1%), *γ*-terpinene (2.0%), caryophyllene oxide (1.6%), thymyl methyl ether (0.9%), *trans*-*β*-caryophyllene (0.9%), humulene epoxide II (0.7%), and terpinen-4-ol (0.7%).

UIS: Industrial University of Santander (Bucaramanga, Colombia).

**Table 2:** Minimal inhibitory concentration values of the most active EOs studied and of some commercial terpenes against *Candida* species.

Species	MIC (µg/mL)								Source
	AMB		ITC		FLC		CSF		
	Range	GM	Range	GM	Range	GM	Range	GM	
*C. albicans* ATCC 64550	0.03–0.12	0.04	0.5–1	0.7	4–8	5.6	0.25–0.5	0.35	Collection
*C. albicans* ATCC 10231	0.03–0.12	0.04	0.03–0.125	0.06	4	4	0.12–0.25	0.18	Collection
*C. parapsilosis* ATCC 22019	0.06	0.06	0.25–0.50	0.35	0.5–1	0.70	1	1	Collection
*C. krusei* ATCC 6258	0.12–0.25	0.15	0.12–0.50	0.28	8	8	1	1	Collection
*C. tropicalis* ATCC 750	0.06–0.12	0.11	0.06–0.25	0.12	1–2	1.4	0.25–0.5	0.35	Collection
*C. tropicalis* ATCC 200956	1–2	1.41	>16	>16	>64	>64	0.5	0.5	Collection
*C. glabrata* LMDM 34	0.06–0.12	0.06	1	1	2–4	2.8	8	8	Collection
*C. metapsilosis* MUM 15.12	<0.03	<0.03	0.06–0.12	0.09	1–2	1.4	1	1	Collection
*C. orthopsilosis* MUM 17.13	<0.03	<0.03	0.12	0.12	1	1	1	1	Collection
*C. lusitaniae* MUM 17.08	0.03	0.03	0.03–0.06	0.04	0.5	0.5	1	1	Collection
*C. auris* CDCB11903	0.06–0.12	0.07	0.06–0.12	0.04	1–2	1.4	0.5	0.5	Collection
*C. auris* Ca 1	0.12–0.25	0.20	0.03–0.12	0.06	4	4	0.5	0.5	Subcutaneous tissue
*C. auris* Ca 9	0.12–0.5	0.25	0.03–0.12	0.06	4	4	0.5	0.5	No data available
*C. auris* Ca 10	0.12–0.25	0.18	0.06–0.12	0.09	4	4	0.5–1	0.7	Pleura tissue
*C. auris* Ca 32	0.12–0.5	0.19	0.25–0.50	0.35	8–16	11.3	0.5	0.5	Parietal pleura
*C. auris* Ca 41	1	1	0.03–0.12	0.06	2	2	0.5	0.5	Groin smear
*C. auris* Ca 45	1–2	1.41	0.06–0.12	0.04	2	2	0.5	0.5	Axillary smear
*C. auris* Ca 46	1–2	1.4	0.06–0.12	0.04	2–4	2.8	0.5	0.5	Axillary smear
*C. auris* Ca 17	0.06–0.25	0.15	0.12–0.5	0.25	32	32	0.5	0.5	Urine culture
*C. parapsilosis* Synlab 406	0.06–0.25	0.15	0.5–1	0.70	8–16	11.3	2	2	Blood culture

GM: geometric mean; LMDM: Laboratorio de Micología y Diagnóstico Molecular; MUM: Micoteca da Universidade do Minho; AMB: amphotericin B; ITC: itraconazole; FLC: fluconazole; CSF: caspofungin.

**Table 3:** Minimal inhibitory concentration values of the most active EOs studied and of some commercial terpenes against non-*C. auris* species.

EO Code/Terpene	GM—Range MIC (μg/mL)					
	*C. krusei* ATCC 6258	*C. tropicalis* ATCC 200956	*C. parapsilosis* Synlab 406	*C. metapsilosis* MUM 17.13	*C. orthopsilosis* MUM 15.12	*C. lusitaniae* MUM 17.08
2206	256	181 (128–256)	16	128	128	181 (128–256)
0008	256	181 (128–256)	16	128	128	181 (128–256)
0010	256	128	22.6 (16–32)	90.5 (64–128)	128	128
0018	256	128	22.6 (16–32)	90.5 (64–128)	128	90.5 (128–64)
0019	NA	181 (128–256)	64	128	128	181 (128–256)
Thymol	181 (128–256)	90.5 (64–128)	64	128	128	90.5 (64–128)
Carvacrol	256	128	45.3 (32–64)	128	128	181 (128–256)
Perillyl alcohol	NA	181 (128–256)	90.5 (64–128)	256	256	181 (128–256)
*p*-Cymene	181 (128–256)	181 (128–256)	256	256	256	256
Limonene	32	64	64	64	16	22.6 (16–32)

GM: geometric mean; NA: non active.

**Table 4:** Minimal inhibitory concentration values of the most active EOs studied and of some commercial terpenes against *C. auris*.

EO Code/Terpene	GM—Range MIC (μg/mL)								
	*C. auris* CDC B11903	*C. auris* Ca 1	*C. auris* Ca 9	*C. auris* Ca 13	*C. auris* Ca 32	*C. auris* Ca 17	*C. auris* Ca 41	*C. auris* Ca 45	*C. auris* Ca 46
2206	128	256	256	256	64	128	128	128	90.5 (64–128)
0008	128	256	256	256	181 (128–256)	128	128	128	90.5 (64–128)
0010	64	NA	256	256	64	90.5 (64–128)	64	64	64
0018	64	256	181 (128–256)	256	128	64	64	64	64
0019	128	256	181 (128–256)	256	90.5 (64–128)	128	128	128	128
Thymol	64	181 (128–256)	128	128	64	64	64	64	64
Carvacrol	90.5 (64–128)	181 (128–256)	256	181 (128–256)	64	128	90.5 (64–128)	90.5 (64–128)	64
Perillyl alcohol	256	256	128	256	128	256	128	256	256
*p*-Cymene	256	NA	256	256	128	256	256	256	256
Limonene	64	64	22.6 (16–32)	22.6 (16–32)	22.6 (16–32)	64	16	16	16

GM: geometric mean; NA: non active.

**Table 5:** The 50% cytotoxic concentration (CC_50_) and SI values of the most active EOs and commercial terpenes.

EO Code/Terpene	HaCaT Cells Mean CC_50_ (µg/mL)	SI Range (CC_50_/MIC)
2206	788.0	3.0–49.2
0008	877.9	3.4–54.8
0010	903.6	3.5–56
0018	665.9	2.6–41
0019	354.7	1.4–5.5
Thymol	427.5	3.3–6.7
*p*-Cymene	831.2	3.2–5.5
Carvacrol	410.7	1.6–12.8
Limonene	400.5	3.1–50
Perillyl alcohol	400.7	1.6–6.25

## Data Availability

Data are contained within the article.

## Sample Availability

Samples of the compounds are not available from the authors.
